# Dual roles of interleukin-33 in cognitive function by regulating central nervous system inflammation

**DOI:** 10.1186/s12967-022-03570-w

**Published:** 2022-08-16

**Authors:** Xiuqin Rao, Fuzhou Hua, Lieliang Zhang, Yue Lin, Pu Fang, Shoulin Chen, Jun Ying, Xifeng Wang

**Affiliations:** 1 Department of Anesthesiology, The Second Affiliated Hospital of Nanchang University, Nanchang, 330006 Jiangxi China; 2 Key Laboratory of Anesthesiology of Jiangxi Province, 1# Minde Road, Nanchang, 330006 Jiangxi People’s Republic of China; 3 Department of Neurology, The First Affiliated Hospital of Nanchang University, Nanchang, 330006 Jiangxi China; 4 Department of Anesthesiology, The First Affiliated Hospital of Nanchang University, Nanchang, 330006 Jiangxi China

**Keywords:** Interleukin-33, Suppression of tumorigenicity 2, Neuroinflammation, Cognitive function, Microglia, Astrocytes, Apoptosis, Synaptic plasticity

## Abstract

With the advent of an aging society, the incidence of dementia is increasing, resulting in a vast burden on society. It is increasingly acknowledged that neuroinflammation is implicated in various neurological diseases with cognitive dysfunction such as Alzheimer’s disease, multiple sclerosis, ischemic stroke, traumatic brain injury, and central nervous system infections. As an important neuroinflammatory factor, interleukin-33 (IL-33) is highly expressed in various tissues and cells in the mammalian brain, where it plays a role in the pathogenesis of a number of central nervous system conditions. Reams of previous studies have shown that IL-33 has both pro- and anti-inflammatory effects, playing dual roles in the progression of diseases linked to cognitive impairment by regulating the activation and polarization of immune cells, apoptosis, and synaptic plasticity. This article will summarize the current findings on the effects IL-33 exerts on cognitive function by regulating neuroinflammation, and attempt to explore possible therapeutic strategies for cognitive disorders based on the adverse and protective mechanisms of IL-33.

## Introduction

Cognitive impairment is a symptom of numerous neurological diseases, such as Alzheimer’s disease (AD), multiple sclerosis (MS), ischemic stroke, traumatic brain injury (TBI), and central nervous system (CNS) infections, typically affecting learning and memory, language, executive function, complex attention, and social cognition [1–3]. Cognitive decline affects the daily life of the patient and has a significant negative impact on the family and society [4]. Therefore, it is crucial to investigate the specific mechanisms of cognitive impairment and to attempt to find possible therapeutic targets.

**Fig. 1:**
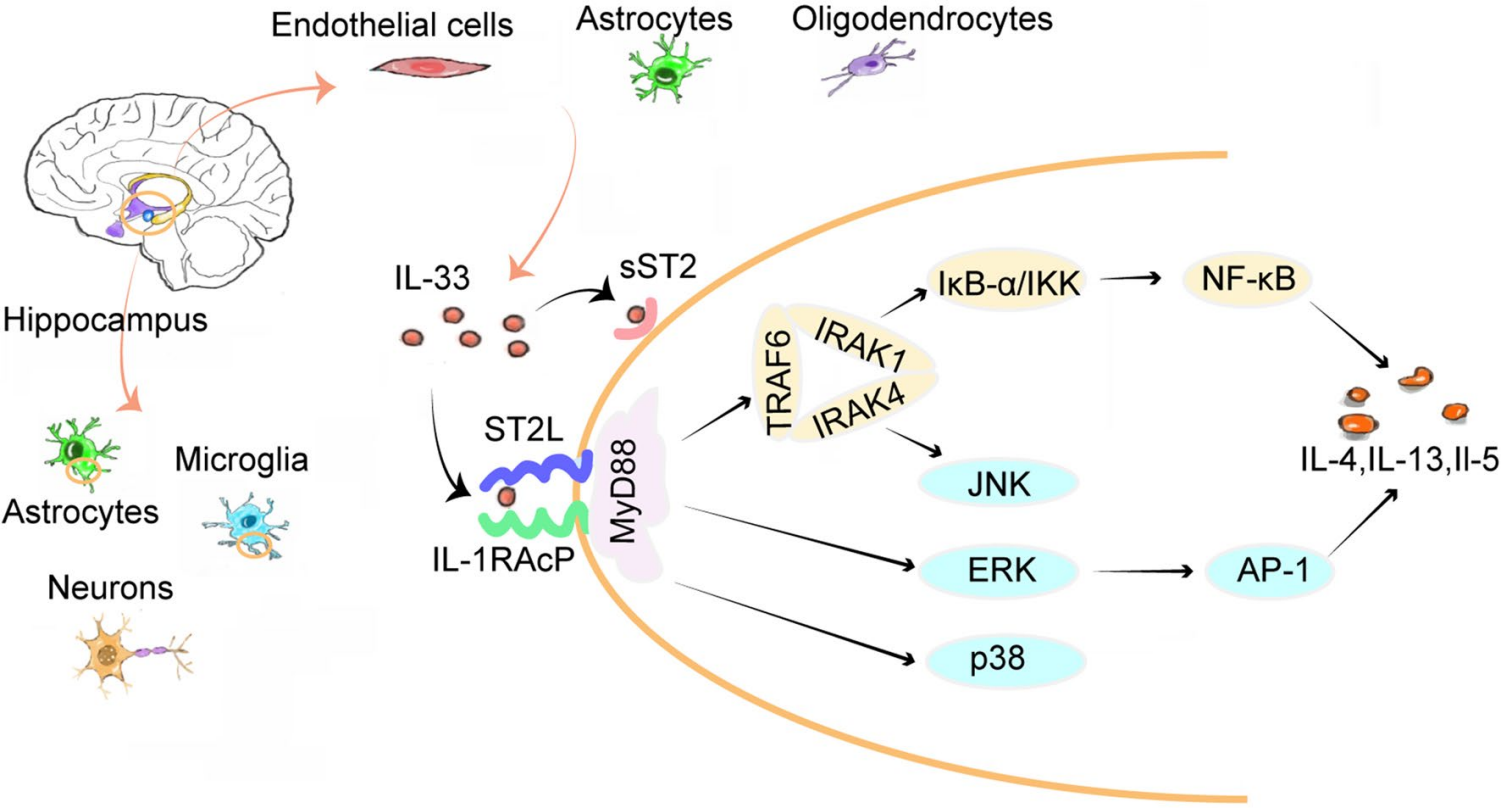
IL-33 intracellular pathways. (1) IL-33 is mainly produced by endothelial cells, astrocytes, and oligodendrocytes. Its receptor complex ST2L/IL-1RAcP is constitutively expressed by microglia, astrocytes, and neurons in the hippocampus, which is associated with cognitive function. (2) IL-33 binds to the cell surface receptor complex ST2L/IL-1RAcP and induces MyD88 recruitment to the complex. (3) sST2 as an inducible receptor competes with ST2L to bind IL-33 and inhibits IL-33/ST2L signaling pathway-related effects. (4) Receptor-associated MyD88 facilitates the activation of IRAK1 and IRAK4 with TRAF6 recruitment. IRAKs induce the activation of IκB-α and IKK resulting in NF-κB activation. (5) Activated MyD88 also induces the phosphorylation of kinases ERK, and p38, along with IRAKs-induced JNK resulting in AP-1 activation. (6) NF-κB and AP-1 induce the production of the Th2-associated cytokines, including IL-4, IL-5, and IL-13

Interleukin-33 (IL-33) is a recently discovered multifunctional member of the IL-1 family [5, 6], that is constitutively expressed in the CNS tissues of both humans and mice [6], mainly in endothelial cells, astrocytes [6], and oligodendrocytes [7, 8] (Fig. 1). Suppression of tumorigenicity 2 (ST2), the specific receptor of IL-33 [5], is mainly expressed in microglia and astrocytes [6], but also in mast cells, T helper 2 cells (Th2), oligodendrocytes, macrophages, and neurons [9]. IL-33 and ST2 have different expression levels and specific roles in different regions of the CNS [10]. The finding that ST2 was highly expressed in the hippocampus [6], known as a higher center for cognitive functions such as learning and memory [11], has led to the discovery of a role of IL-33 in cognitive function [12]. Neuroinflammation is widely considered an important pathophysiological mechanism underlying cognitive impairment in various neurological disorders [13, 14]. IL-33 binds to its receptor on ST2-expressing cells to induce the production of cytokines, chemokines, and potentially neurotoxic substances, such as IL-4, IL-13, IL-5, IL-6, IL-10, interleukin-1beta (IL-1β), tumor necrosis factor-alpha (TNF-α), interferon-γ (IFN-γ), nitric oxide (NO), and radical oxygen species (ROS) [6, 15]. These mediators play pivotal roles in neuroinflammation or neuroprotection [16–18] and are significantly associated with the state of cognition [19–22].

IL-33 has been proposed to play a role in the progression of several neurological diseases by modulating neuroinflammation [23–26]. The association of IL-33 with cognitive impairment was also demonstrated in studies of patients and animal models of neuroinflammation-related diseases [2, 3, 27, 28]. IL-33 administration reversed memory deficits in APP/PS1 mice, an animal model of AD, which was associated with decreased inflammation [2]. Conversely, IL-33-deficient rats exhibited impaired social novelty recognition [29]. However, an opposing view was also put forward, according to which IL-33 administration in mice led to a spatial memory performance deficit associated with an increase of inflammatory markers in the hippocampus [30]. Therefore, we deduce that IL-33 may have opposite effects on cognition through divergent regulation of inflammation in the CNS. This review used the keywords IL-33, cognitive function, neuroinflammation, and related diseases to select relevant literatures from the PubMed database. In this review, we summarize the regulatory mechanisms of IL-33 related to CNS inflammation in some neurological diseases (Tables 1, 2). Finally, the possible mechanisms driving the dual effects of IL-33 on cognitive function in neurological disorders are discussed, particularly in AD, MS, TBI, ischemic stroke, and CNS infections.

**Table 1:** Roles of IL-33 in brain neurological diseases

Diseases/models	IL-33/ST2 level	Mechanism	Function	References
Damage				
AD and MCI patients	IL-33(↑) ST2(↑)	Higher levels of apolipoprotein E ε4 and phosphorylated tau are indeed associated with cognitive decline	Patients expressing IL-33 preserve their cognitive function	[66]
MS patients	IL-33(↑)	Inhibits CNS myelination	Involves in the pathogenesis of all MS	[8, 118, 147, 148]
EAE mice	IL-33(↑) ST2(↑)	Enhances Th1/Th17 response Inhibits Treg response	Promotes EAE	[113]
HIV-infected cells	IL-33(↑) ST2L(↑)	Leads to neuroinflammation Dys-regulates synaptic function and apoptosis	Promotes HIV	[135]
ECM	IL-33(↑)	Orchestrates an amplification loop between IL-1β and IL-33 in microglia and oligodendrocytes to exacerbate neuroinflammation	Exacerbates neurological and cognitive defects	[3]
Protect				
TBI human and mice	IL-33(↑)	Promotes recruitment of microglia and release of pro-inflammatory mediators	Promotes TBI	[150]
APP/PS1 mice	IL-33(↑) sST2(↓)	Reverses synaptic plasticity impairment Promotes microglia polarization toward anti-inflammatory M2 Promotes microglia phagocytic activity to Aβ uptake	Ameliorates AD and cognitive decline	[47, 80]
EAE mice	IL-33(↑) ST2(↑)	Switches a predominantly pathogenic Th17/Th1 response to Th2 activity Promotes microglia polarization toward anti-inflammatory M2 Suppresses the activation of astrocytes and microglia	Attenuates EAE	[78, 84, 112]
ECM	–	Reduces pro-inflammatory cytokine and chemokine Drives the expansion of ILC2 to produce Type-2 cytokines Leads to the polarization of the anti-inflammatory M2 and expands Treg	Prevents the development of ECM	[79]
RNS mice	IL-33(↓) ST2(↓)	Inhibits apoptosis, ER stress, and autophagy Reverses the up-regulation of IL-1β and TNF-α levels	Attenuates RNS-induced neurobehavioral disorders and spatial learning and memory deficits	[28, 85]
ICH mice/rats	IL-33(↓) ST2L(↑)	Suppresses the expression of pro-inflammation cytokines IL-1β and TNF-α Promotes microglia M2 polarization Suppresses apoptotic and autophagic activation	Alleviates ICH-induced neurological deficits, neuronal degeneration, cell death, and neurobehavioral deficits	[87, 88]
Stroke mice/MCAO mice	–	Inhibits Th1/Th17 response Enhances Treg response Induces immune-shift of Th cells from Th1 to Th2 response Promotes microglia polarization toward anti-inflammatory M2	Provides neuroprotection	[90, 100, 115]
TBI mice	IL-33(↑) ST2L(↓)	Inhibits autophagy, ERS, and apoptosis Prevented TBI-induced increase of IL-1β and TNF-α levels to inhibit neuroinflammation Promotes the polarization of M2 microglial and type-2 phenotype cytokines production	Mitigates TBI-induced motor function outcome, spatial learning, and memory deficits	[86, 91]
Stroke patients and mice models	IL-33(↑) sST2(↑)	Increases M2-type microglia and induces IL-4 secretion Reduces astrocytes activation	Reduces ischemia-induced sensorimotor deficits	[116]

**Table 2:** Mechanisms of IL-33 and its related-cytokines

Cytokines	Receptors	Pathways	Results
IL-33 as a transcription factor	–	Interferes with the binding of p65 to κB consensus binding sites	Dampens the pro-inflammatory signaling pathway
High level of IL-33	ST2L	NF-κB signaling pathway Activates and recruits astrocytes Promotes microglia polarization to M1 Reinforces Th17 and Th1 cell functions	Induces the production of IL-6, IL-8, IL-17, IL-1β, TNF-α, IFN-γ, CCL2, GMF, NO, ROS Negative effect on synaptic plasticity
Low level of IL-33	ST2L	NF-κB, AP-1, and MAP kinases p38, JNK, and ERK1/2 signaling pathway Promotes microglia polarization to M2 Promotes Th cells polarization to Th2 and Treg Inhibits Th cells polarization into Th1 and Th17 Inhibits microglia polarization into M1	Induces the production of IL-4, IL-5, IL-13, IL-10 Decreases the release of IL-17, IL-6, IL-12, IL-18, IFN-γ, IL-1β, TNF-α, CCL2, ROS, and NO Inhibits ERS, autophagy, and apoptosis Exerts a protective effect on synaptic plasticity
IL-33	sST2	Compete with ST2 for IL-33	Inhibits the effect of the IL-33/ST2L signaling pathway
IL-4, IL-5, IL-13	–	Inhibit NO production through STAT6 Increase BDNF in hippocampal astrocytes	Inhibit neuroinflammation Promote learning-dependent synapse formation
TNF-α, IL-1β, IL-18, and IL-6, ROS, NO	–	NF-κB signaling pathway A-calcium–calmodulin-dependent protein kinase II, MAPK, and ERK pathways	Induce neuroinflammation Induce ERS, autophagy, and apoptosis Impair the synaptic plasticity

### The intracellular IL-33/ST2 signaling pathway

IL-33 is localized in the nucleus as a transcription factor and extracellularly as a cytokine [9]. IL-33, ST2L, and IL-1 receptor accessory protein (IL-1RAcP) are highly expressed in brain cells, in particularly microglia, astrocytes, and neurons [5, 6, 25]. As a cytokine, IL-33 is upregulated and released upon inflammatory stimulation after injury and is thought to play roles in neuroinflammatory diseases via its binding to the ST2 receptor [6, 7, 31, 32]. The IL-33/ST2 signaling pathway participates in a complex network of multicellular interactions between the immune and nervous systems in the CNS [33] and is also central for infection-induced cognitive impairments [3]. However, it is worth noting that the IL-33/ST2 signaling pathway has dual roles in a range of infectious and inflammatory diseases [34], mediating both pathological immune responses and anti-inflammation neuronal responses [10, 31, 35].

**Fig. 2:**
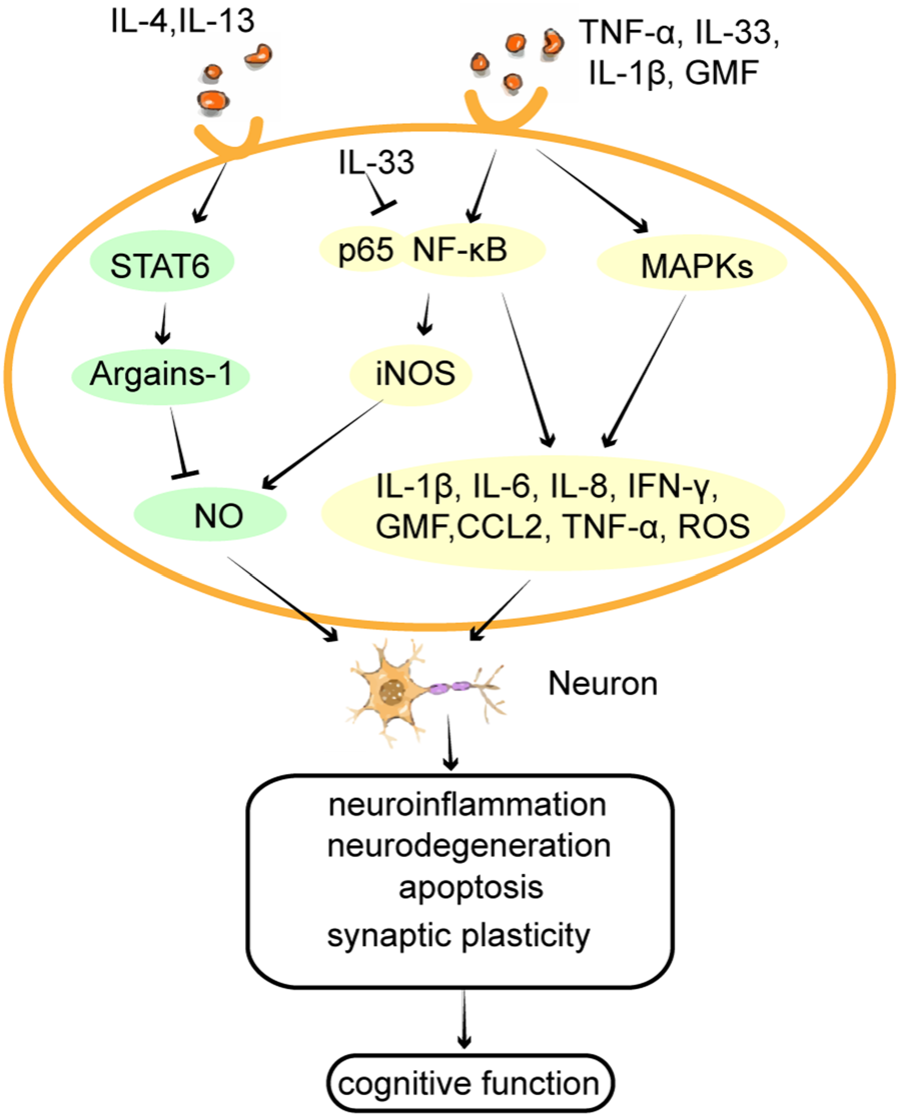
Inflammatory cytokines intracellular pathways. (1) IL-4 and IL-13 induce arginase I expression via STAT6 activation and arginase I efficiently competes with iNOS for substrate l-arginine, causing a decreased output of NO in astrocytes and microglia. (2) High levels of IL-33 and other pro-inflammatory molecules may activate the NF-κB and MAPKs signaling pathways, which induces numerous pro-inflammatory cytokines, chemokines, and neurotoxic mediators, such as IL-1β, IL-6, IL-8, IFN-γ, TNF-α, CCL2, GMF, NO and ROS in astrocytes and microglia. (3) IL-33 binds to p65 in the nucleus as a transcription factor, blocking the conjugation of p65 to the NF-κB transcription factor. It directly inhibits the nuclear translocation of NF-κB, which inhibits the NF-κB downstream pro-inflammatory signaling pathway and suppresses the inflammatory response. (4) These IL-33-induced inflammatory mediators act on neurons and modulate neuroinflammation, neurodegeneration, apoptosis, and synaptic plasticity, ultimately, cognitive function

In this part, the signaling pathways through which IL-33 exerts its protective effects on cognition are summarized. In IL-33^−/−^ mice (IL-33 gene deleted mice) and myeloid differentiation factor 88 (MyD88)-deficient mice, the phenomenon of long-term potentiation (LTP; one of the most commonly used models of measuring hippocampal memory) was abolished, and both the acquisition and retention latencies in the Morris water maze test (MWM; one of the most common behavioral methods for assessing spatial learning and memory) were increased [36]. This indicates that the IL-33/ST2 pathway may regulate spatial learning and memory by targeting MyD88 [36]. As a member of the IL-1 family, IL-33 acts through a signaling pathway similar to other cytokines in the family [5]. ST2 has been described as a negative regulator of Toll-like receptor-IL-1 receptor signaling, and functions as an important effector molecule of T helper type 2 responses [5, 37, 38]. In detail, IL-33 binds a cell membrane receptor composed of ST2L and IL-1RAcP, inducing the recruitment and activation of MyD88 to the receptor complex. Subsequently, the stimulated receptor-associated MyD88, as an adaptor protein, facilitates the recruitment and activation of interleukin-1 receptor-associated kinase 1 (IRAK1), interleukin-1 receptor-associated kinase 4 (IRAK4), and tumor necrosis factor receptor-associated factor 6 (TRAF6) [5, 39]. IRAKs further induce the downstream phosphorylation of IκB-α and IκB kinase (IKK), triggering the nuclear translocation of nuclear transcription factor-κB (NF-κB) and activation of C-Jun N-terminal kinase (JNK). In addition, the activated MyD88 induces the phosphorylation of extracellular signal-regulated kinases (ERK) and p38 mitogen-activated protein kinase (MAPK) [40], which constitute the MAPK signaling pathways along with JNK, leading to the triggering of activator protein-1 (AP-1). Finally, NF-κB and AP-1 transcription factors may induce the production of Th2-associated cytokines, including IL-4, IL-5, and IL-13 [41, 42] (Fig. 1). IL-4 and IL-13 induce arginase I expression via signal transducer and activator of transcription 6 (STAT6), and arginase I efficiently competes with inducible nitric oxide synthase (iNOS) for the substrate L-arginine, causing a decrease of NO generation in astrocytes and microglia [43] (Fig. 2). These Th2-associated downstream responses might balance the inflammatory Th1 responses to protect neurons from NO damage. The poor performance of IL-4- and IL-13-deficient mice in the MWM is accompanied by a decrease of brain-derived neurotrophic factor (BDNF) in hippocampal astrocytes. BDNF can promote learning-dependent synapse formation, which contributes to synaptic longevity and plasticity as well as the formation of neuronal circuits [44]. This indicates that IL-13 and IL-4 may also enable astrocytes to produce BDNF to aid spatial learning and memory consolidation [45]. In addition, the binding of intracellular IL-33 to NF-κB transcription factors was also found to contribute to the down-regulation of NF-κB and anti-inflammatory signaling in human HEK293RI cells (human embryonal kidney cells) in vitro. IL-33 interferes with the binding of p65 to κB consensus binding sites and reduces NF-κB/p65-triggered gene expression to dampen this pro-inflammatory signaling pathway [46]. Similarly, recombinant IL-33 was also observed to significantly reduce the nuclear translocation of NF-κB and disrupt its downstream pro-inflammatory mechanisms in monocytes derived from patients with AD and mild cognitive impairment (MCI) in vitro [47] (Fig. 2).

However, when released in excess of the physiological level, IL-33 may act as a pro-inflammatory cytokine to activate inflammatory cells, including astrocytes, microglia, and mast cells, in the CNS. IL-33 may activate the NF-κB signaling pathway, which upregulates numerous pro-inflammatory genes [48] and was implicated in the up-regulation of pro-inflammatory cytokines, chemokines, and neurotoxic mediators, such as IL-1β, IL-6, IL-8, IFN-γ, TNF-α, chemokine ligand 2 (CCL2), glia maturation factor (GMF), NO and ROS, resulting in inflammatory responses and cognitive dysfunction in neurodegenerative diseases [34, 49–52] (Fig. 2). Thus, we speculate that IL-33 binding to ST2 may affect cognition through pro- or anti-inflammatory effects, which are mediated by the NF-KB signaling pathways in ST2-expressing cells, especially microglia and astrocytes.

### IL-33 and glial cells

#### Effects of IL-33 on glial cells activation and proliferation

IL-33 was found to promote the proliferation, recruitment, and activation of astrocytes and microglia of mice in vivo or in vitro [6, 15, 30]. The activation and proliferation of microglia and astrocytes are hallmarks of neurodegenerative and CNS autoimmune disorders [25]. As the main inflammatory cells in the CNS, microglia and astrocytes secrete several pro-inflammatory factors, such as IL-33, TNF-α, IL-1β, IL-18, IL-6, ROS, and NO, leading to neuroinflammation, which is thought to be associated with cognitive impairment [11, 15, 40, 53–55]. Numerous studies found that IL-33 promotes the release of pro-inflammatory mediators and neurotoxic substances from activated microglia and astrocytes in vitro, such as IL-18, IL-6, IL-1β, GMF, CCL2, TNF-α, and NO [15, 40, 56]. The high-level expression of pro-inflammatory cytokines and chemokines in the CNS is thought to contribute to neuroinflammation and neuronal injury, leading to cytokine-induced cognitive impairment [13, 30, 57]. For instance, it has been reported that GMF contributes to cognitive deficits in mice, which may be associated with the stimulation of the MAPK and NF-κB signaling pathways, resulting in the up-regulation of pro-inflammatory cytokines such as TNF-α and IL-1β in astrocytes [57–59]. CCL2-mediated neurotoxic effects cause neuronal damage and neurodegeneration, which may lead to cognitive decline in several CNS diseases, such as stroke, AD, and MS [60, 61].

**Fig. 3:**
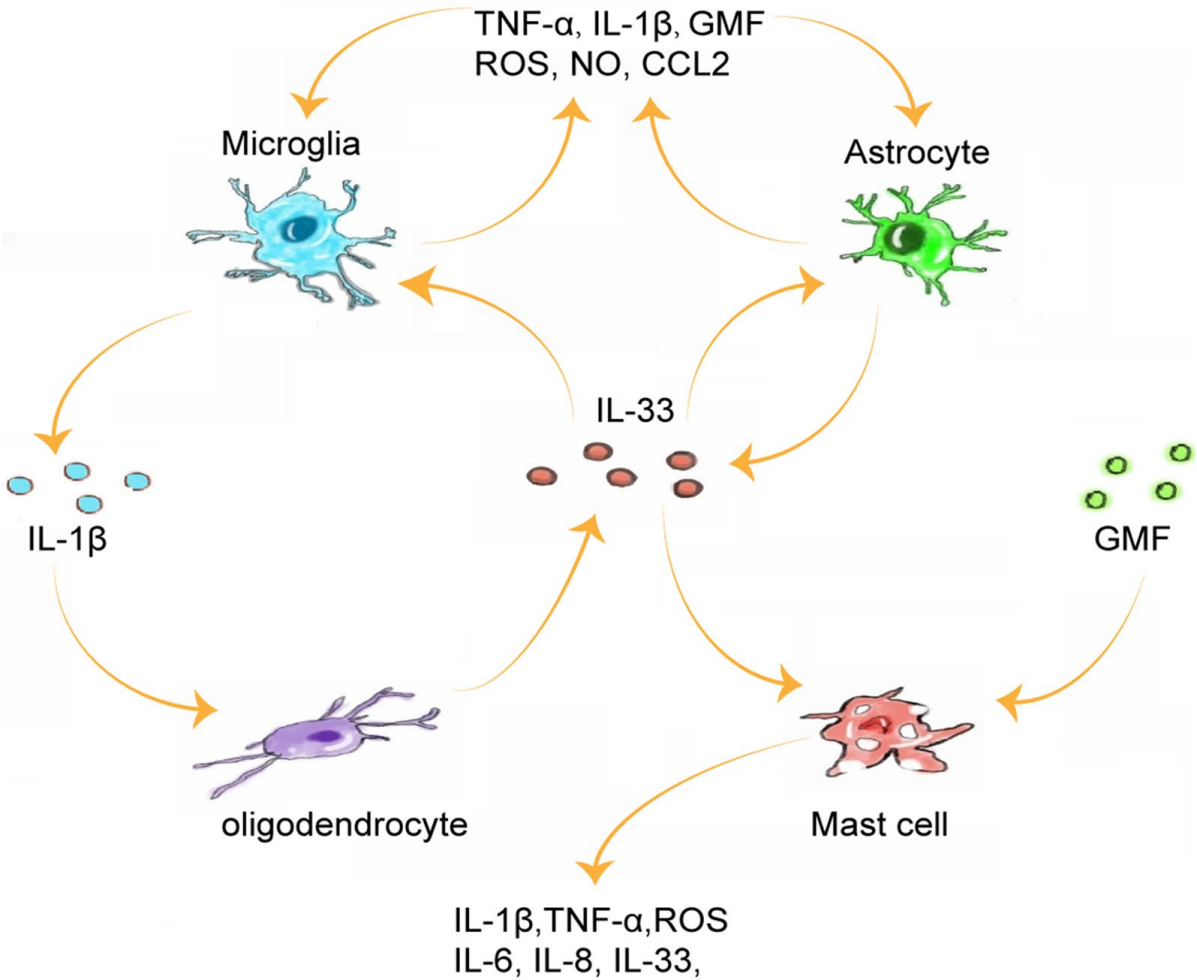
The amplification loops of IL-33 in CNS. (1) Autocrine loop in astrocytes: IL-33 is inducible by inflammatory stimuli in astrocytes. IL-33 induces the release of pro-inflammatory cytokines from astrocytes. Pro-inflammatory cytokines stimulate astrocytes to release more IL-33. (2) IL-33 secretion by astrocytes activates other immune cells to release pro-inflammatory mediators in the brain, and pro-inflammatory mediators further promote the release of IL-33 from these cells. (i) Amplification loop between astrocytes and microglia: pro-inflammatory mediators produced by IL-33-activated microglia, such as IL-1β, TNF-α, GMF, ROS, NO, and CCL2, which in turn activate microglia to secret more pro-inflammatory mediators and secret high levels of IL-33 from astrocytes and microglia. (ii) Amplification loop between GMF and IL-33 in astrocytes and mast cells: GMF induces the release of inflammatory mediators IL-33, ROS, and CCL2 in astrocytes and mast cells. IL-33 also augments the release of GMF-mediated IL-1β, TNF-α, ROS, and CCL2 in astrocytes and mast cells. (iii) Amplification loop between IL-1β and IL-33 in microglia and oligodendrocytes: IL-33 induces the production of IL-1β in microglia through IL-33/ST2 pathway, and IL-1β activates oligodendrocytes in turn to secret high levels of IL-33

In this review, we sum up that IL-33 and other pro‑inflammatory mediators may form autocrine and paracrine amplification loops between astrocytes and other immune cells, further exacerbating CNS inflammation and leading to cognitive impairment [3]. It was recently reported that astrocytes released IL‑33 as an alarmin following inflammatory stimulation, and IL‑33 also induced the production of pro‑inflammatory factors in astrocytes, suggesting that it formed an autocrine feedback loop in astrocytes [32]. IL-33 and pro-inflammatory mediators produced by IL-33-activated astrocytes may activate other immune cells, such as microglia and mast cells, inducing a more severe inflammatory response, which in turn leads to the high-level secretion of IL-33 and pro-inflammatory mediators [40, 62, 63]. For instance, in mouse astrocytes and mast cells cultured in vitro, GMF was found to induce the release of inflammatory mediators IL-33, ROS, and CCL2 [40, 62], and IL-33 also augmented GMF-mediated IL-1β, TNF-α, ROS, and CCL2 release [40, 62, 63]. Thus, there may be an amplification loop between GMF and IL-33 in astrocytes and mast cells. A study in a mouse model of experimental cerebral malaria (ECM), induced by Plasmodium berghei ANKA (PbA) infection, has shown that the expression of IL-33 and ST2 increased in astrocytes and oligodendrocytes in the hippocampus of infected animals. PbA-infection-induced early short-term and spatial memory defects in wild-type mice, which was proportional to the IL-33 level, while ST2-deficient mice did not develop a cognitive deficit [3]. IL-33 induced the production of IL-1β in microglia via the IL-33/ST2 pathway, and IL-1β in turn activated oligodendrocytes to secret high levels of IL-33 in vitro [3]. Thus, the amplification loop between IL-1β and IL-33 in the CNS may contribute to plasmodium-induced cognitive deficits [3] (Fig. 3).

Beta-amyloid peptide (Aβ) and tau proteins are the main components of amyloid plaques (APs) and neurofibrillary tangles (NFTs), which are the main pathological features of the AD brain. Aβ and tau plaques activate innate immunity, thus leading to the production and release of substantial amounts of pro-inflammatory mediators (NO and ROS) and cytokines (IL-1β, IL-10, IL-33, and TNF-α). This leads to neuroinflammation [64] and synaptic damage in AD neurons [65], which are associated with cognitive function [66, 67]. In the brains of human patients and AD model mice, IL-33 and ST2 are respectively highly expressed in astrocytes in close proximity to APs and NFTs, whereby IL-33 may exacerbate the release of pro-inflammatory molecules from astrocytes and contribute to Aβ-induced cognitive deficits [51, 59]. A study of tissue sections from human TBI and mouse models of CNS injury has shown that the expression of IL-33 in the brain was elevated along with the up-regulation of IL-1β and TNF-α secretion by microglia. However, ST2 deficiency reduced the number of infiltrating microglia and pro-inflammatory cytokines in the mouse brain following TBI [68]. Thus, the IL-33/ST2 signaling pathway may contribute to the cognitive impairment typical of neurological diseases at least in part by promoting the proliferation, recruitment, and activation of astrocytes and microglia, which release pro-inflammatory cytokines.

#### Effects of IL-33 on microglia polarization and phagocytosis

**Fig. 4:**
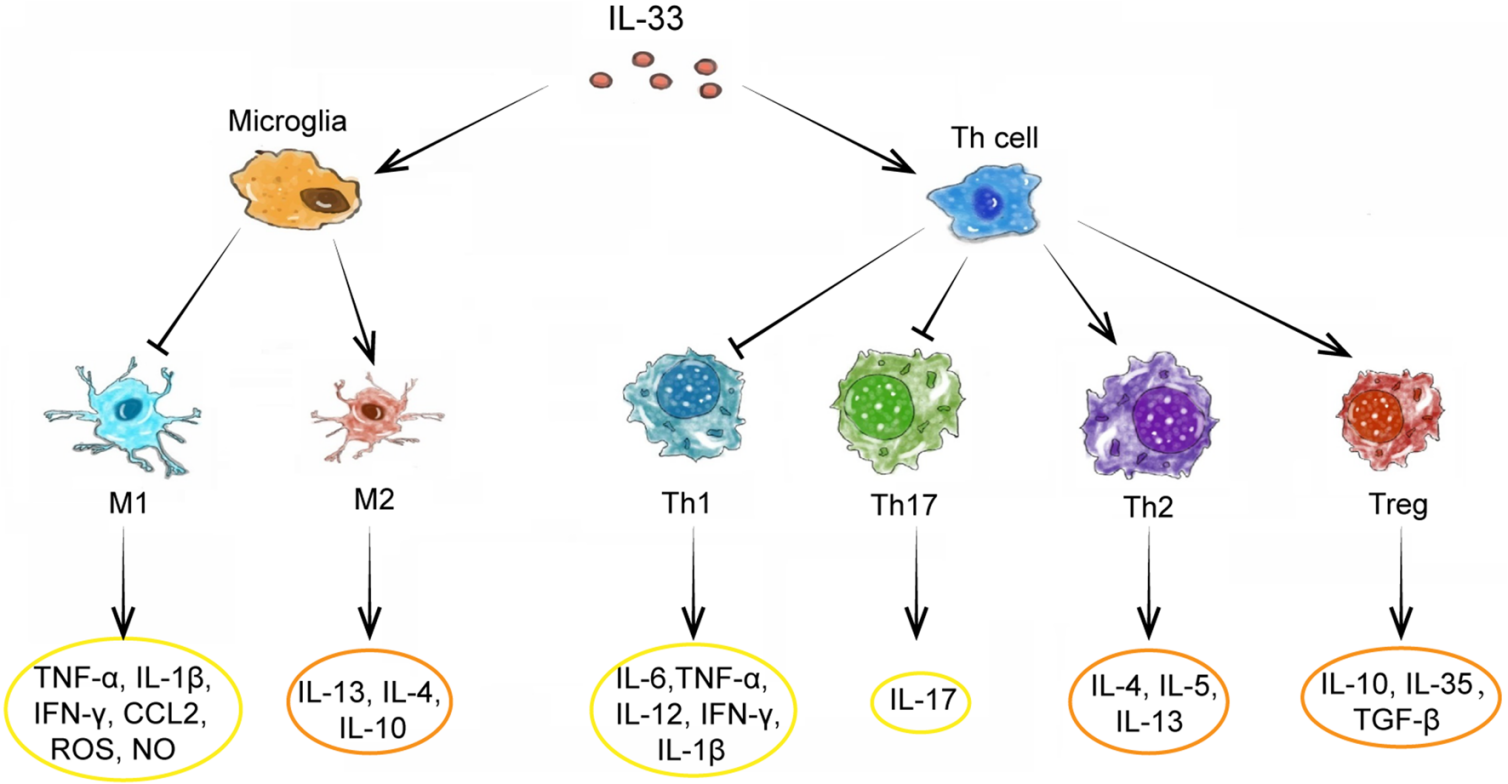
Effect of IL-33 on microglia and Th cell polarization. (1) IL-33 inhibits the transformation of (i) microglia into M1-type to produce TNF-α, IL-1β, IFN-γ, ROS, and NO. (ii) Th cells into Th1 to produce IL-6, IL-12, IL-1β, IFN-γ, and TNF-α. (iii) Th cells into Th17 to produce IL-17. (2) IL-33 promotes the conversion of (i) microglia into M2-type to produce IL-4, IL-13, and IL-10. (ii) Th cells into Th2 to produce IL-4, IL-5, and IL-13. (iii) Th cells into Treg to produce IL-10, IL-35, and TGF-β. (3) The overall effects of IL-33 on the transformation of microglia and Th cells are to inhibit the release of pro-inflammatory cytokines and promote the release of anti-inflammatory cytokines. These regulatory molecules exert their effects on neurons, regulating neuroinflammation and cognitive function

In a comprehensive analysis, IL-33 may exert a protective effect by promoting the polarization of microglia to the anti-inflammatory type (Fig. 4), which may play a role when the inflammatory responses are limited. Microglia are derived from mononuclear phagocytes and are the characteristic immune cells in the brain, which can be activated into two phenotypes including the classically-activated phenotype (M1) and alternatively-activated phenotype (M2) [69]. The polarization of microglia into M1 or M2 phenotypes depends on the cytokine milieu in the tissue [64]. Microglia develop into the pro-inflammatory M1 phenotype in response to type 1 cytokines and microbial products, such as IFN-γ, IL-2, and LPS [69], while the anti-inflammatory M2 phenotype is induced by type 2 cytokines including IL-4 [70], IL-21 [71], and IL-13 [69]. M1 microglia can promote the release of pro-inflammatory mediators and neurotoxic substances, such as TNF-α, IL-1β, IFN-γ, IL-18, IL-6, CCL2, ROS, and NO, which are associated with impaired phagocytic function, leading to nerve damage and death [69]. Conversely, M2 microglia can release anti-inflammatory mediators, such as IL-13, IL-4, and IL-10, to inhibit the destructive type 1 immune response, leading to increased phagocytic function [72–74], thus removing cell debris from the brain and playing a therapeutic role in CNS diseases [75, 76]. Microglia express a significant level of ST2 [6, 77, 78], and are the targets of IL-33 in the brain [30]. In contrast to the pro-inflammatory effects promoted by IL -33 described in “Effects of IL-33 on glial cells activation and proliferation” section, IL-33 was also observed to drive the expansion of type-2 innate lymphoid cells (ILC2) that produce type-2 cytokines (IL-4, IL-5, and IL-13), leading to the polarization of anti-inflammatory M2 microglia [79].

Synthesizing the present researches, IL-33 has been shown to promote the anti-inflammatory effects of M2 microglia to protect cognition in multiple disease models. In APP/PS1 mice, pro-inflammatory genes including IL-1β and IL-6 were significantly induced in the brain cortex, and the exogenous IL-33 administration significantly suppressed the increased expression of these pro-inflammatory genes by polarizing microglia toward an anti-inflammatory phenotype, thereby modulating the immune response [2, 80, 81]. Macrophages infiltrating the CNS in concert with activated resident microglia play a major role in the inflammatory neurodegenerative process of MS and experimental allergic encephalomyelitis (EAE; a widely used animal model for human MS) [82, 83]. An increase of M2 cells suppressed ongoing severe EAE and promoted the recovery, while imbalance towards M1 induced the relapse of EAE in mice. Moreover, macrophages from IL-33-treated EAE mice showed increased polarization toward the M2 phenotype, and the EAE mice that had received macrophages from IL-33-treated mice exhibited significantly reduced disease severity and accelerated recovery [84]. In ECM, IL-33 also induced anti-inflammatory M2 microglia polarization through expansion of ILC2 to suppress the pro-inflammatory response, thereby preventing the development of ECM [79]. A study of recurrent neonatal seizures (RNS) has shown an evident up-regulation in the expression of TNF-α and IL-1β as well as a remarkable increase of NF-κB activity in both the cortex and hippocampus of the RNS brain, which were associated with cognitive impairment. These changes in RNS rats were prevented by IL-33 pretreatment [85]. Similar changes were observed in a TBI mouse model [86]. In the mouse intracerebral hemorrhage (ICH) model, IL-33 treatment promoted the transformation of microglia to M2, and suppressed the expression of pro-inflammation cytokines IL-1β and TNF-α in the brain tissues, thereby alleviating ICH-induced brain damage and neurobehavioral deficits [87, 88]. In rat models of ischemic stroke, the expression of IL-33 and ST2 was elevated, which suppressed neuronal damage by promoting microglial polarization toward the M2 phenotype and cytokine production associated with the M2 macrophage-like microglial immune phenotype [33, 89–91]. Conversely, blocking the IL-33/ST2 pathway led to a transformation of microglia into the M1 phenotype, which increased neuroinflammation and injury [89]. The neuroprotective effect of the cannabinoid WIN 55,212-2 following CO poisoning might also be attributed to its stimulating action on hippocampal microglia, where it promotes M2 polarization via IL-33/ST2 signaling pathway [75]. The available studies in these disease models have demonstrated that IL-33/ST2 pathway might inhibit the inflammatory response and reduce cognitive impairment by promoting the polarization of microglia toward the anti-inflammatory M2 phenotype, which may be a reliable therapeutic strategy [2, 30, 80, 88, 92].

IL-33 has been shown to enhance the phagocytosis of mouse microglia cultured ex vivo [6], possibly by inducing microglia to convert to the M2 type. In AD, high levels of Aβ and phosphorylated tau protein are associated with cognitive impairment [66, 67]. M2 microglia eliminated Aβ accumulation by enhancing Aβ phagocytosis, clearance, and degradation [25, 93, 94]. IL-33 mRNA and protein levels were lower in the brains of AD patients and mouse models of AD compared with healthy individuals [2], and patients with high IL-33 expression had better cognitive function [66]. IL-33 administration increased the numbers of phagocytic microglial cells around APs, and the amounts of soluble Aβ were significantly reduced in the cortices of IL-33-treated APP/PS1 mice, preventing cognitive decline [2, 24, 80]. Conversely, IL-33 deficiency caused neurodegeneration and deposition of abnormal tau protein, including hyper-phosphorylated, paired helical fragment, and insoluble tau in the cerebral cortex and hippocampus of aging IL-33^−/−^ mice, which was accompanied by the early onset of aging-related AD-like impairment of cognition and memory [95]. IL-33 may therefore have a protective effect against aging-induced cognitive impairment by promoting the phagocytotic activity of microglia. According to the available literature, IL-33 may have therapeutic potential in the preservation of cognitive function by polarizing microglia toward the M2 phenotype, which exhibits enhanced phagocytosis to increase the elimination of Aβ and tau protein.

### Effects of IL-33 on the polarization of helper T cells (Th cells)

The cytokines derived from T helper 1 cells (Th1) and T helper 17 cells (Th17), most prominently IFN-γ and IL-17, promote inflammation and subsequent secondary brain damage [96], while Th2-derived cytokines IL-4 and IL-13 have been shown to preserve spatial learning ability and memory by reducing inflammation and promoting neurogenesis [45]. A positive association between the serum levels of IL-17 and the montreal-cognitive assessment scores was noted in patients with schizophrenia, particularly in visuospatial and executive functioning, as well as in language functioning and delayed recall [97]. Regulatory T cells (Tregs) may also alleviate cognitive decline in mouse models of AD, possibly by constraining neuroinflammation through the upregulation of IL-10 [98]. The imbalance between Th1/Th17 and Th2/Tregs in the CNS was thought to be crucial for the neuroinflammatory and cognitive impairment in multiple neurological brain disorders, such as MS, brain injury, and ECM [79, 99–101]. IL-33 is a potent inducer of type 2 immunity [102]. Th2 [103], mast cells, and ILC2 [104] express ST2 at significant levels, linking IL-33 to type 2 immune responses [105]. Mast cells and Th2 can respond to IL-33 stimulation, which leads to a significant increase in the production of Th2-associated anti-inflammatory cytokines, including IL-4, IL-5, and IL-13 [41, 42, 106, 107]. IL-33 can also induce Tregs to produce anti-inflammatory cytokines, including IL-10, IL-35, and TGF-β [108–110]. In addition, IL-33 was found to decrease the secretion of Th17/Th1-associated pro-inflammatory cytokines, including IL-17, IL-6, IL-12, IFN-γ, IL-1β, and TNF-α [106, 108]. Hence, IL-33 may promote the transformation of Th cells into anti-inflammatory Th2 and Tregs, while also inhibiting their polarization into Th1 and Th17 (Fig. 4). This promotes the secretion of anti-inflammatory factors while at the same time inhibiting the secretion of pro-inflammatory factors, which may reduce neuroinflammation and neuronal damage.

The effect of IL-33 on the polarization of Th cells has also been demonstrated in several disease models, which are summarized in this review. IL-33 or ST2 knockout exacerbated spinal cord demyelination and EAE progression by promoting the development of pathogenic effector Th cells, including Th1 and Th17, while suppressing Th2 cells and Treg cells in the CNS of EAE mice [78, 111–113]. Conversely, IL-33-treated EAE mice exhibited significantly reduced amounts of serum IL-17 and IFN-γ, along with increased levels of IL-5 and IL-13 compared with control mice [84]. Hence, IL-33 may play a protective role in EAE by promoting Th2 and inhibiting Th1/Th17. Mice with induced cerebrovascular occlusions, such as middle cerebral artery occlusion (MCAO) and unilateral common carotid artery occlusion, are commonly employed as experimental models of ischemic stroke [100, 114]. In mouse models of ischemic stroke, memory deficits in novel object recognition tasks after acute brain ischemia were associated with increased brain levels of the pro-inflammatory cytokines TNF-α and IL-1β [114]. In MCAO mice, administration of recombinant IL-33 promoted Th2-type effects following focal ischemic stroke, resulting in increased plasma levels of IL-4, IL-5, and IL-13, as well as attenuating the stroke-induced increases of the pro-inflammatory cytokines IFN-γ, IL-17, IL-1β and IL-6 [100, 115, 116]. In addition, IL-33 treatment increased the number of Tregs in the ischemic brain as well as the levels of IL-10, IL-35, and TGF-β in serum and brain tissues of MCAO mice [109, 110]. Thus, IL-33 treatment attenuated the severity of brain injury caused by dysregulated inflammation [100] and may mitigate cognitive impairment after focal cerebral ischemia. Similarly, in the oxygen–glucose deprivation neural cell model, IL-33 treatment effectively increased the concentration of IL-4 and TGF-β, while significantly reducing the level of IL-17 [117]. Additionally, in a murine model of ECM, IL-33 induced the release of Th2 cytokines IL-4, IL-5, and IL-13, as well as the expansion of Tregs, while suppressing the pro-inflammatory response, which alleviated cognitive impairment and prevented ECM development [3, 79]. These studies indicate that IL-33 may be at least partly responsible for an induced immune-shift from Th1 to Th2 response and suppressing the Th17 immune response to exert anti-inflammatory effects and reduce cognitive impairment after CNS injury.

However, some studies also showed increased concentrations of IL-33 in patients with MS and mouse models of EAE [113, 118]. In EAE mice, IL-33 treatment enhanced IFN-γ and IL-17 production by Th cells, while anti-IL-33 treatment reversed this increased and suppressed the development of EAE [113]. IL-33 may be involved in the pathogenesis of the EAE and MS through the reinforcement of Th17 and Th1 cell functions [113, 118], possibly correlated with excessive IL-33.

### Effects of IL-33 on autophagy and apoptosis

This review explores the possibility that IL-33 affects cognitive function by regulating cellular autophagy and apoptosis. Autophagy is a lysosome-dependent cellular degradation program. Moderate autophagy is an essential pathway for maintaining cellular homeostasis, but deregulated autophagy causes neuronal cell death, hippocampal shrinkage, and ultimately leads to a loss of synaptic plasticity [119]. Similarly, apoptosis is an evolutionarily conserved cell death pathway. Moderate apoptosis is necessary for normal development and maintenance of tissue homeostasis [120], while excessive neuronal apoptosis caused by excessive levels of pro-inflammatory molecules will accelerate neurodegeneration and cognitive impairment [14, 121, 122]. Endoplasmic reticulum stress (ERS) also leads to calcium dysregulation, which induces cell death pathways associated with autophagy and apoptosis, including activation of initiator caspases and the modulation of Bcl-2 family members [123]. Abnormal neuroinflammation, autophagy, and apoptosis are crucial factors of neuronal cell damage, which leads to the onset and progression of cerebrovascular disorders and cognitive dysfunction [124]. High levels of autophagy-related molecules such as Beclin-1, upregulation of pro-apoptotic proteins such as caspase-3, cleaved-caspase-3 (CC-3), Drp1, and Bax, as well as the downregulation of anti-apoptotic proteins such as Bcl-2 in the brain are associated with the decline of learning and memory function in a rat model of iron overloaded vascular dementia [125].

Some studies have shown that IL-33 may protect cognitive function by inhibiting excessive autophagy and apoptosis [85–87, 126]. For example, IL-33 significantly inhibited the trauma-induced upregulation of the ERS-related protein glucose-regulated protein 78 (GRP-78; localized in the lumen of the ER, and involved in the folding and assembly of proteins in the ER), pro-apoptotic proteins caspase-3, CC-3, and Bax, as well as the autophagy-related molecule Beclin-1, while also downregulating the anti-apoptotic protein Bcl-2 in the hippocampus of TBI, ICH and MCAO model mice [86, 87, 127]. IL-33 pretreatment remarkably improved the motor function and MWM test scores in these mouse models. Notably, these positive effects of IL-33 can be blocked by anti-ST2 treatment [86, 87]. Thus, IL-33/ST2 signaling may mitigate TBI- and ICH-induced cognitive impairments through the inhibition of ERS, autophagy, and apoptosis [86, 87, 128]. Similarly, RNS induced significant increases in GRP-78, Drp1, and CC-3 expression and a decrease in Bcl-2 expression in the hippocampus, leading to neurobehavioral disorders, along with deficits of spatial learning and memory. Notably, these effects could be mitigated by IL-33 pretreatment in RNS mice [85].

### Effects of IL-33 on synaptic plasticity

Continuous synaptic remodeling in the hippocampus is essential for the encoding and retaining of learning and memory [129]. Synaptic plasticity, the ability of neurons to alter the structure and strength of synapses, is important for memory formation and consolidation [130]. Synaptic dysfunction is manifested in several ways, including loss of synapses, reduced synaptic function, and impairment of synaptic plasticity, which are the result of CNS insults and prolonged inflammation linked to cognitive deficits [14, 65, 131]. Synaptic impairment in its various forms is not only a hallmark of late-stage neurodegenerative diseases, such as Parkinson’s disease [132] and AD [133], but also a feature of the early progression of dementia [65]. Cytokines such as TNF-α, IL-1β, IL-18, and IL-6, as well as other molecules such as ROS, NO, and prostaglandin E2, are overexpressed during the neuroinflammatory process. These signals deeply influence neurotransmitter release and post-receptor signal transduction mechanisms, ultimately affecting synaptic function through a-calcium–calmodulin-dependent protein kinase II, MAPK, and ERK pathways or by directly influencing the synaptic memory processes [134]. At low physiological levels, these immune mediators might be essential for the induction and maintenance of neuroplasticity, but their overexpression during a neuroinflammatory process might result in neurodegeneration and the impairment of synaptic plasticity [134].

In this review, we summarize that the synaptic plasticity of neurons is also influenced by IL-33 in several studies [24, 135–137]. Neuron-microglia communication through IL-33 is required for precision memory at remote time points, and IL-33 promotes microglial functions to optimize experience-dependent neural circuit plasticity, suggesting that IL-33 may play a role in memory consolidation [136]. IL-33 treatment significantly reversed the LTP impairment and contextual memory deficits of APP/PS1 mice, while also improving habituation to a new environment, which confirmed that IL-33 treatment reversed the reduction of synaptic plasticity in the hippocampus and cognitive deficits in APP/PS1 mice [2, 24]. The cornu ammonis 1 (CA1) sub-region constitutes the primary output node of the hippocampus, which is thought to be essential for most hippocampus-dependent memories [138]. IL-33 administration promoted the formation of functional excitatory synapses in hippocampal CA1 neurons in vivo, whereas conditional knockout of IL-33 in CA1 astrocytes decreased the number of excitatory synapses [137]. Importantly, blockade of IL-33 by intracerebroventricular administration of its decoy receptor inhibited homeostatic synaptic plasticity in CA1 pyramidal neurons in vivo, thereby impairing spatial memory formation in mice [137]. Thus, IL-33 may play a key role in learning and memory by enhancing synapse formation. The effect of IL-33 on synaptic plasticity modulation in HIV-infected human astrocyte cultures was indicated by an inverse correlation of IL-33 and myocyte enhancer factor 2C (MEF2C), a transcription factor that regulates synaptic function in the hippocampal region. A low concentration of IL-33 (120 ng/L) may exert a protective effect on synaptic plasticity with an increased MEF2C, while the higher dose of IL-33 (480 ng/L) led to a significant down-regulation of MEF2C and had a negative effect on synaptic plasticity. It was therefore speculated that the accumulation of IL-33 negatively impacted the synaptic plasticity, which may potentially be explained by the pro-inflammatory feedback effect of IL-33 [55, 135].

## Conclusions

Although many studies investigated the expression and roles of IL-33 in CNS diseases and resulting cognitive dysfunction since IL-33 was originally reported in 2005 [5], there is still no consensus regarding the distribution and the exact effect of IL-33 in the basal ganglia, cerebellum, cerebral cortex, hippocampus, and midbrain of humans [17]. IL-33 induced the phagocytosis and expression of pro-inflammatory cytokines in microglia in a dose-dependent manner [6]. IL-33 concentrations were significantly correlated with the Glasgow coma scale scores following TBI [23]. IL-33 regulation of synaptic function in HIV infection is also dose-dependent [135]. We suggest that the concentration of IL-33 at the site of CNS lesions may be associated with the complicated role of IL-33. IL-33 may be a neuroprotective factor at appropriate concentrations [24, 47], while high concentrations of IL-33 in the neuropathological lesions of the brain may exacerbate neuroinflammation and cognitive decline [51]. ST2 has 2 major cognate isoforms: the transmembrane form (ST2L) and the soluble form (sST2). The transmembrane form of ST2L is the main functional receptor, but the sST2 receptor can act as a decoy to reduce the binding of IL-33 to the ST2L [6, 41, 139]. Accordingly, the binding of IL-33 to sST2 may have opposite effects to those of ST2 by neutralizing and blocking the IL-33/ST2 signaling pathway [47, 139–141]. The levels of IL-33 and sST2 were positively correlated with the cognitive performance of patients with schizophrenia [140]. The serum concentration of sST2 was significantly increased in patients with AD and MCI [2, 47]. Elevated concentrations of plasma sST2 levels were significantly associated with cognitive impairment in acute ischemic stroke patients [142, 143]. Elevated plasma levels of sST2 may contribute to neuronal injury and long-term neurocognitive impairment in older children with cerebral malaria [144]. Its ability to bind two different isoforms of ST2 may at least partly account for the dual roles of IL-33. In contrast to the protective role of IL-33 in EAE [84, 112], treatment with recombinant IL-33 worsened the disease course of EAE in mice [113]. IL-33 has exhibited both damaging and protective properties, possibly based on the period of the disease. IL-33 may have a preventive effect against EAE at the initial stage of the disease. However, after the establishment of EAE, it may have an enhancing role in disease development due to the presence of inflammatory cells in the CNS. Thus, the amount of IL-33, the cells and stage of the disease it acts on, as well as the different receptors it binds to may account for the complexity of its effects [6, 10, 51, 135, 140]. The contradictory results may be attributed to some factors that have yet to be elucidated. For example, one study revealed that sex-determined differences in IL-33 expressions by innate immune cells in response to myelin peptide immunization may influence EAE susceptibility [145]. The specific mechanisms through which IL-33 affects cognition in different neurological diseases still require further research.

The CNS inflammatory response, termed neuroinflammation, is a fundamental response generated to clean up the lesion, limit its area, and protect the CNS. Nevertheless, uncontrolled or prolonged neuroinflammation is potentially harmful and may have cytotoxic effects, aggravating the incidence and severity of the disease [146]. In this review, we summarized the mechanisms of the pro- and anti-inflammatory effects of IL-33 in CNS disorders. The dual effects of IL-33 on cognitive function in CNS disorders may be associated with neuroinflammation. IL-33 regulates both pro- and anti-inflammatory cellular mediators secreted by microglia, astrocytes, and Th cells, influencing inflammation-mediated autophagy, apoptosis, and synaptic plasticity. When inflammation is limited, modest IL-33 levels may limit nerve damage and slow disease progression by promoting the polarization of microglia and Th cells toward anti-inflammatory phenotypes and enhancing the phagocytotic activity of microglia to facilitate the clearance of hazardous substances and necrotic cells. By contrast, prolonged and sustained inflammatory reactions can up-regulate the production of IL-33 and other inflammatory molecules by immune cells, resulting in inflammatory amplification loops in the brain, which may lead to the dysregulation of autophagy, apoptosis, and synaptic plasticity to exacerbate neuronal damage, ultimately leading to cognitive impairment. However, how IL-33 switches between protective and destructive effects remains unclear. If the specific molecules leading IL-33 to switch between pro- and anti-inflammatory effects can be clarified, it may be possible to maximize the anti-inflammatory effects of IL-33 in the CNS by modulating its concentration or targeting specific receptors in specific stages of related diseases to ultimately protect cognitive function. Possible therapeutic strategies aiming to control or treat cognitive impairment in brain neuroinflammation-related diseases need to be verified in the future.

In this review, the mechanisms underlying the dual role of IL-33 on cognitive function in neurological disorders have been discussed, but there are still some insufficiencies that need to be further investigated in the future. Previous studies on the roles of IL-33 in patients have mostly been limited to monitoring changes of serum IL-33 levels during disease progression and in post-mortem tissue sections of patients, which may only suggest a link between IL-33 and symptoms alteration or evolution, while the exact mechanisms were still unclear [8, 66, 118, 147, 148]. Most studies on the roles of IL-33 have been conducted in cultured cells in vitro or animal models. Incubating cells with IL-33 in vitro, site-specific IL-33 injections in mice, and IL-33 or ST2 blockade in mice are routine techniques for studying the roles of IL-33 in different diseases. In vitro, IL-33 treatment may facilitate the clarification of the effects of IL-33 on specific cells, but there are still some limitations. Because of the complexity of cellular responses and homeostasis of organisms, the effects of IL-33 in vivo are not as simple as those observed in cell culture experiments. The effects of IL-33 may be affected by complex cell network interactions, as IL-33 has different effects on different cells, and its receptors are also diverse, as well as the interplay between the extracellular and intracellular forms of this cytokine, which may account for the complex roles of IL-33 in vivo [5, 6, 9, 46, 139, 149]. In addition, most of the current experimental data do not take gender into account, in fact, the effect of IL-33 may be sex-influenced [145]. Current experimental data have suggested that IL-33 may have opposite effects at different stages of disease [112, 113], but research on the specific role of IL-33 at different stages of disease progression is still scarce. In addition, although it has been presented that the effect of IL-33 on cognition is correlated with its concentration [6, 23, 135], there is still a shortage of studies on precise IL-33 concentration gradients. Although mouse models can recapitulate many of the characteristic changes observed in the progression of human diseases, the translation of the experimental data to preclinical and clinical studies should also be the focus of future research. The significance of IL-33 as a possible therapeutic target for the preservation of cognitive function in neurological diseases of the CNS cannot be overstated. Nevertheless, the regulators that control the switching between the therapeutic and damaging effects of IL-33 have not been found yet, and should be studied intensely in the future.

## Data Availability

The data supporting the conclusion of this review have been included within the article.

## Abbreviations

CNS: Central nervous system
AD: Alzheimer’s disease
MS: Multiple sclerosis
TBI: Traumatic brain injury
MCI: Mild cognitive impairment
IL-33: Interleukin-33
ST2: Suppression of tumorigenicity 2
IL-1β: Interleukin-1beta
TNF-α: Tumor necrosis factor-alpha
IFN-γ: Interferon-γ
NO: Nitric oxide
ROS: Radical oxygen species
CCL2: Chemokine ligand 2
GMF: Glia maturation factor
IL-1RAcP: IL-1 receptor accessory protein
LTP: Long-term potentiation
MWM: Morris water maze test
MyD88: Myeloid differentiation factor88
IRAK1: Interleukin-1 receptor-associated kinase 1
IRAK4: Interleukin-1 receptor-associated kinase 4
TRAF6: Tumor necrosis factor receptor-associated factor 6
JNK: C-Jun N-terminal kinase
IKK: IκB kinase
MAPK: Mitogen-activated protein kinase
NF-κB: Nuclear transcription factor-κB
AP-1: Activator protein-1
STAT6: Signal transducer and activator of transcription 6
iNOS: Inducible nitric oxide synthase
BDNF: Brain-derived neurotrophic factor
ECM: Experimental cerebral malaria
PbA: Plasmodium berghei ANKA
Aβ: β-Amyloid peptide
Aps: Amyloid plaques
NFTs: Neurofibrillary tangles
M1 microglia: Classically-activated phenotypes microglia
M2 microglia: Alternatively-activated phenotypes microglia
ILC2: Type-2 innate lymphoid cells
EAE: Experimental allergic encephalomyelitis
RNS: Recurrent neonatal seizures
ICH: Intracerebral hemorrhage
Th cells: Helper T cells
Th1: T helper 1 cells
Th17: T helper 17 cells
Th2: T helper 2 cells
Treg: Regulatory T cells
MCAO: Middle cerebral artery occlusion
ERS: Endoplasmic reticulum stress
CC-3: Cleaved-caspase-3
GRP-78: Glucose-regulated protein 78
CA1: Cornu ammonis 1
MEF2C: Myocyte enhancer factor 2C
ST2L: The transmembrane form ST2
sST2: The soluble form ST2
